# Canadian Workers’ Well-Being During the Beginning of the COVID-19 Pandemic: A Latent Profile Analysis

**DOI:** 10.1007/s41042-023-00142-1

**Published:** 2024-01-04

**Authors:** Tyler Pacheco, Simon Coulombe, Nancy L. Kocovski

**Affiliations:** 1 Department of Psychology, Wilfrid Laurier University, Waterloo, ON Canada; 2 Relief Research Chair in Mental Health, Self-management and Work, Université Laval, Québec City, QC Canada; 3 Department of Industrial Relations, Université Laval, Québec City, QC Canada; 4 VITAM – Sustainable Health Research Centre, Québec City, QC Canada; 5 CERVO Brain Research Centre, Québec City, QC Canada; 6 Centre d’études et d’interventions en santé mentale, Université Laval, Québec City, QC Canada; 7 Centre for the Study of Democratic Citizenship, Montréal, QC Canada

**Keywords:** COVID-19, Well-being, Workers, Latent Profile Analysis, Canada

## Abstract

To explore workers’ well-being during COVID-19, researchers have primarily utilized variable-centered approaches (e.g., regression) focusing on describing workers’ general level of well-being. Given the diversity of factors that may have impacted workers’ well-being during the pandemic, focusing on such well-being trends do not provide sufficient insight into the different lived well-being experiences during the pandemic. Moreover, positive well-being in workers’ general lives and work has been understudied in such complex public health crises. To address these issues, we use latent profile analysis, a person-centered analysis, to explore the diverse well-being realities Canadian workers (employed before COVID-19 or working at the time of the survey) experienced at the beginning of COVID-19. Canadian workers (*N* = 510) were surveyed between May 20-27th, 2020, on positive (meaning in life, flourishing, thriving at work) and negative (distress, stress, impaired productivity, troublesome symptoms at work) well-being indicators, as well as on factors that may be associated with experiencing different well-being profiles. Five well-being profiles emerged: moderately prospering, prospering, moderately suffering, suffering, and mixed. Factors at the self- (gender, age, disability status, trait resilience), social- (marital status, family functioning, having children at home), workplace- (some employment statuses and work industries, financial strain, job security), and pandemic-related (perceived vulnerability to COVID-19, social distancing) ecological levels predicted profile membership. Recommendations for employers, policymakers, and mental health organizations are discussed.

With the coronavirus (COVID-19) pandemic being one of the most significant crises to hit the world, one would assume that the unprecedented circumstances of the pandemic would result in poor mental well-being. However, is this the case? Studies focused on workers’ well-being during pandemics and epidemics have shown that workers, despite multiple stressors associated with such crises, experienced high positive psychological well-being (i.e., prospering) or had experienced it more than negative well-being (Pacheco et al., submitted; e.g., also see: McAlonan et al., 2007). COVID-19-related research, however, has predominately used variable-centered approaches. Using such approaches has left unexplored the diverse well-being realities within working populations. In the current study, we use a form of person-centered analysis to address this limitation.

## Worker Well-Being and the Pandemic

Well-being is often classified pathogenically or salutogenically. The pathogenic camp explores a traditional conceptualization of well-being where mental health is equated to the absence of disability, disease, and premature death (Keyes, 2007). Sahebi et al. (2021), for example, utilized a pathogenic well-being lens and showed that the prevalence of anxiety and depression among healthcare workers in various countries during COVID-19 was 24.94%. The salutogenic camp explores a contemporary conceptualization rooted in positive psychology, which views well-being as the “presence of positive states of human capacities and functioning in cognition, affect, and behavior” (Keyes, 2014, p. 179). The Two Continua Model (Keyes, 2005) combines these perspectives and suggests that well-being is a complete state characterized by the absence of mental health concerns and the presence of positive well-being (Westerhof & Keyes, 2010). Although both well-being facets should be explored, positive outcomes are understudied (Waters et al., 2022).

Some evidence sheds light on workers’ positive well-being in their general lives. For example, healthcare workers in special COVID-19 emergency wards in Pakistan had a mean life satisfaction score of 3.59 during the pandemic (Rafiq et al., 2022), indicating moderate to moderately high life satisfaction. Bassi et al. (2021) report that 33.40% of healthcare workers in Lombardy, Italy, were flourishing. The other 66.60% reported moderate mental health (57.70%) and languishing (8.90%).

### Workers’ Work-Related Well-Being

Manifestations of well-being are also unique to different life domains (e.g., work). In a scoping review and meta-analysis, for example, Ghahramani et al. (2021) report that approximately half of the healthcare workers in diverse countries experienced burnout during COVID-19. In contrast, positive well-being may, for example, manifest as a sense of thriving at work (e.g., Kleine et al., 2019). Canadian workers had moderate levels of thriving at work one to two weeks after social distancing measures were implemented (Pacheco et al., 2020). Similarly, Chinese non-managerial restaurant employees had an average score of 5.68 on a positive well-being at work measure (Huo, 2021), indicating a moderately high level of the construct. These findings suggest a counterintuitive phenomenon: positive well-being was frequently experienced during the COVID-19 pandemic.

### Differential Experiences in Well-Being

Risk and resilience factors found at different ecological levels may affect the degree to which positive and negative well-being are experienced during pandemics. Whereas risk factors increase individuals’ vulnerability to daily stressors, resilience factors protect them against the effect of daily stressors (Diehl et al., 2012). Reminiscent of Bronfenbrenner’s (1986) Ecological System Theory, factors exist at different ecological levels ranging in proximity to workers. Pacheco et al. (submitted) show that commonly explored proximal (e.g., age, gender) and distal (e.g., occupation, risk/exposure, knowing someone infected or killed by the virus) factors are significantly related to worker well-being during such crises. There are many factors, however, that need attention (e.g., immigration status, non-healthcare work industries).

This ecological approach is aligned with the third wave of positive psychology, which is multidisciplinary, recognizes the complexity of well-being, and goes beyond the individual (Lomas et al., 2021; Wissing, 2022). Guided by the third wave of positive psychology, Wissing (2022) posits that a multi-, inter-, or transdisciplinary approach is required during challenging circumstances (e.g., COVID-19) to understand the complexity of different well-being-related dimensions (e.g., psychological, sociological). Using a third-wave perspective provides more freedom to merge theories that help holistically understand the impacts of COVID-19 on worker well-being. As such, theories in public health (social determinants of mental health; Alegría et al., 2018), positive psychology (Resilience Theory, Pan & Chan, 2007; The Two Continua Model, Keyes, 2005; Westerhof & Keyes, 2010), developmental and community psychology (e.g., Ecological Systems Theory, Bronfenbrenner, 1986; Jason et al., 2016) and industrial-organizational psychology (e.g., precarious work, Allan et al., 2021) were used to explore how diverse Canadian workers’ well-being was affected early in the pandemic.

### Towards Person-Centered Analyses

Variable-centered statistical approaches (e.g., regression) dominantly used in social and psychological sciences do not accurately explore heterogeneity within samples. Instead, such analyses use a few variables, and trends regarding full samples tend to be published (Howard & Hoffman, 2018). Although parsimonious, these approaches focus on overall samples or very broad groups and do not capture the diverse realities that workers were experiencing.

Person-centered approaches, such as latent profile analysis (LPA), can help capture richness of different well-being realities workers were experiencing early in the pandemic. However, there is a paucity of research using person-centered approaches to explore workers’ holistic well-being during such crises. Person-centered approaches can be used to inductively identify and describe the distinct subpopulations present within samples of workers across a set of relevant indicators of well-being during COVID-19. Researchers can then use characteristics associated with workers to see which workers are more likely to be represented in these different well-being “realities,” thus helping to identify risk and resilience factors. Seen in studies using LPA (e.g., Babb et al., 2022; Harju et al., 2021), person-centered approaches can provide a holistic understanding of workers’ well-being during public health crises for researchers. Using three well-being indicators, Harju et al. (2021) showed that workers in France and the UK experienced one of five well-being profiles (moderately positive, languishing, flourishing, mixed feelings and apathetic) during the first COVID-19 lockdown. Left unexplored are work-specific well-being indicators (e.g., impaired productivity, thriving at work), and how they can be explored simultaneously with general life indicators of well-being to obtain a holistic sense of workers’ well-being during COVID-19.

### The Present Study

Aligned with the Two Continua Model (Keyes, 2005; Westerhof & Keyes, 2010), it is of importance to explore workers’ negative (pathogenic) and positive (salutogenic) well-being in their general lives and at work. To accomplish this, we used diverse well-being indicators in the well-being and workplace literature. To capture workers’ negative well-being in their general lives, we used indicators of distress and stress. Distress is often used as a dependent variable in medical and psychological research (Olsen et al., 2006), and is characterized by negative emotional states (McKenzie & Harris, 2013). Whereas some refer to emotional distress as stress, others view stress as adaptive reactions to disturbances (Schneiderman et al., 2005). It is only when stress responses are severe, repetitive, or prolonged that they lead to negative mental outcomes (e.g., depression, anxiety) (Chu et al., 2022). Flourishing and meaning in life were used as positive general life well-being indicators. Flourishing is a key indicator of positive well-being as it encompasses experiencing positive emotions and functioning well psychologically and socially (Mjøsund, 2021). Sense of meaning in life, derived from Viktor Frankl (1963), encompasses comprehension (i.e., a network of schemas making a meaning framework for life) and purpose (i.e., self-concordant long-term life aspirations that motivate relevant activity) (Steger, 2012). As factors of a presenteeism scale, troublesome symptoms at work and impaired productivity were adopted as work-specific negative well-being indicators. Together, these indicators explore workers’ experiences of mental health concerns (e.g., depressive symptoms) at the workplace, as well as their performance and functioning at work. Thriving at work was used as a work-specific positive well-being indicator, and encompasses feelings of vitality and learning at work (Liu et al., 2021). Previous research has shown that moderate relationships do exist between these well-being indicators (e.g., Coulombe et al., 2020; Pacheco et al., 2020; Um-e-Rubbab, 2022). In addition to the theoretical differences between these well-being indicators (e.g., Keyes, 2005) and the literature showing the importance of exploring a wide breadth of work-related well-being indicators (Fisher, 2014), these moderate relationships illustrate the empirical distinctiveness of these indicators. As these indicators do not always covary with one another, we include each as predictors of the well-being realities workers were experiencing early in the pandemic.

Using LPA, this study aimed to determine:

1. Profiles of workers’ (employed when COVID-19 started or working at the time of the survey) well-being across negative (i.e., distress, stress, impaired productivity, troublesome symptoms at work) and positive (i.e., meaning in life, flourishing, thriving at work) well-being indicators.
2. Associations of profile membership with factors regarding the workers (e.g., gender, trait resilience), their relationships (e.g., marital status, family functioning), employment (e.g., employment status, work industry), and COVID-19 (perceived vulnerability to COVID-19, socially distancing).

## Methods

### Participants

The larger project this study is nested in included three surveys that explored workers’ experiences through time. The first survey (Time 0) was conducted one to two weeks after Canada implemented social distancing measures (March 20-27th, 2020). Time 1 occurred two weeks (April 3rd-10th, 2020) following Time 0; Time 2 was conducted two months (May 20-27th, 2020) following Time 0. Time 2 data were used for this study as we were interested in workers’ well-being after they had some time to adjust. As workers had been adapting for two months, Time 2 data was the most optimal data to use than the other two timepoints. Whereas most participants at Time 2 had been recruited at Time 0, in which there were 1,194 participants, 13.79% used in the present study joined the study at Time 1 or 2. These additional participants at Time 1 and 2 may be a result of participants sharing the survey link with other people in their networks. These additional participants reported that they had work experience, met the remaining inclusion criteria outlined below, and thus were retained.

To participate in the survey, workers had to be at least 18 years old, a Canadian resident, working at least 20 h per week before the COVID-19 pandemic, and able to read English. Before data cleaning, Time 2 contained 521 workers. Participants (*n* = 11) were excluded if they failed or did not answer more than 50% of the attention checks. After this exclusion was applied, our final sample was 510. A sample size of 500 is recommended to ensure that LPA accurately identified the number of profiles in the data (Spurk et al., 2020). Our sample exceeded this recommendation. Table 1 contains the demographic breakdown of our final sample. As seen in the table, the social media subsample had a larger proportion (%) of workers who (were): women or a gender minority, single, in a common law relationship, divorced, in an other romantic relationship, had no children at home, resided in New Brunswick or Yukon, or had one or more disabilities. More workers in the social media subsample did not report their race. Regarding individuals’ work, the social media subsample had a larger proportion (%) of workers: not employed but looking, laid off temporarily or indefinitely, or working in food or healthcare industries. Workers social distancing also had a larger proportion in the social media subsample. The social media subsample also had workers who were younger and experienced more financial strain. The Qualtrics subsample had a larger proportion (%) of workers who (were): men, married, separated, widowed, had one or more children at home, resided in Alberta, British Columbia, Manitoba, Newfoundland and Labrador, Nova Scotia, Ontario, Québec, or Saskatchewan, an immigrant or born in Canada, or had no disabilities. Regarding individuals’ work, the Qualtrics subsample had a larger proportion (%) of workers: working full-time or working in manufacturing. Workers not socially distancing were also found in a larger proportion in the Qualtrics subsample. Across the demographic variables, the effect sizes were mainly small, except for gender which had a medium effect size and age which had a large effect size.

**Table 1:** Characteristics of the total sample (*N* = 510) and broken down by workers sampled from social media (*n* = 212) and qualtrics (*n* = 298)

Variables	Total	Social Media	Qualtrics	*t*^*c*^*or* χ^2 *d*^*Statistic*	*Effect Size* ^*e*^
	*Frequency (%)* *or Mean (SD)*	*Frequency (%)* *or Mean (SD)*	*Frequency (%)* *or Mean (SD)*		
**Age (in Years)**	42.75(12.10)	34.66(10.54)	46.68(10.80)	-11.08*** ^*c*^	-1.12
**Gender**				92.40*** ^*d*^	0.46
Men Women Other Missing/Prefer not to say (%)	170(33.30) 268(52.50) 5(0.10) 13.10	11(5.20) 129(60.80) 5(2.40) 31.60	159(53.40) 139(46.60) 0(0.00) 0.00		
**Marital Status**				39.05*** ^*d*^	0.28
Single Common law Married Separated Divorced Widowed Other	160(31.40) 69(13.50) 221(43.30) 18(3.50) 25(4.90) 7(1.40) 10(2.00)	83(39.20) 38(17.90) 62(29.20) 7(3.30) 11(5.20) 2(0.90) 9(4.20)	77(25.80) 31(10.40) 159(53.40) 11(3.70) 14(4.70) 5(1.70) 1(0.30)		
**Number of Dependents in Household**				6.04** ^*d*^	0.11
None One or more	347(68.00) 163(32.00)	157(74.10) 55(25.90)	190(63.80) 108(36.20)		
**Race**				4.19* ^*d*^	0.10
Caucasian/White Racial minority Missing/Prefer not to say (%)	376(73.70) 61(12.00) 14.30	130(61.30) 13(6.10) 32.50	246(82.60) 48(16.10) 1.30		
**Residing Province or Territory**				25.36** ^*d*^	0.24
Alberta	44(8.60)	14(6.60)	30(10.10)		
British Columbia	66(12.90)	24(11.30)	42(14.10)		
Manitoba New Brunswick Newfoundland and Labrador	20(3.90) 15(2.90) 6(1.20)	5(2.40) 8(3.80) 2(0.90)	15(5.00) 7(2.30) 4(1.30)		
Northwest Territories Nova Scotia Nunavut Ontario Prince Edward Island	0(0.00) 31(6.10) 0(0.00) 206(40.40) 0(0.00)	0(0.00) 11(5.20) 0(0.00) 75(35.40) 0(0.00)	0(0.00) 20(6.70) 0(0.00) 131(44.00) 0(0.00)		
Québec Saskatchewan Yukon Missing (%)	34(6.70) 19(3.70) 2(0.40) 13.10	4(1.90) 0(0.00) 2(0.90) 31.60	30(10.10) 19(6.40) 0(0.00) 0.00		
**Born in Canada**				11.80*** ^*d*^	0.16
Yes No Missing (%)	356(69.80) 87(17.10) 13.10	130(61.30) 15(7.10) 31.60	226(75.80) 72(24.20) 0.00		
**Has One or More Disabilities**				34.87*** ^*d*^	0.28
Yes No Missing/Prefer not to say (%)	55(10.8) 383(75.10) 14.10	37(17.50) 105(49.50) 33.00	18(6.00) 278(93.30) 0.70		
**Financial Strain**	1.90(0.80)	2.07(0.80)	1.79(0.79)	3.74*** ^*c*^	0.35
**Employment Status** ^b^					
Employed full-time	380(74.50)	127(59.90)	253(84.90)	40.74*** ^*d*^	0.28
Employed part-time On Mental health/addiction leave	43(8.40) 1(0.20)	26(12.30) 1(0.50)	17(5.70) 0(0.00)	6.90 ^*d*^ 1.41 ^*d*^	- -
Retired Not employed but looking Not employed nor looking Receiving disability support	3(0.60) 14(2.70) 5(1.00) 2(0.40)	1(0.50) 11(5.20) 3(1.40) 2(0.90)	2(0.70) 3(1.00) 2(0.70) 0(0.00)	0.08 ^*d*^ 8.12** ^*d*^ 0.71 ^*d*^ 2.82 ^*d*^	- 0.13 - -
Receiving social assistance Temporarily laid off Indefinitely laid off	2(0.40) 70(13.70) 20(3.90)	0(0.00) 46(21.70) 15(7.10)	2(0.70) 24(8.10) 5(1.70)	1.43 ^*d*^ 19.48*** ^*d*^ 9.58** ^*d*^	- 0.20 0.14
**Industry of Work** ^a b^					
Construction Education Food Healthcare Manufacturing Services Transportation Other (e.g., automotive) Missing (%)	24(4.70) 72(14.10) 25(4.90) 82(16.10) 30(5.90) 68(13.30) 18(3.50) 192(37.60) 6.10	7(3.30) 35(16.50) 18(8.50) 42(19.80) 3(1.40) 29(13.70) 4(1.90) 77(36.30) 9.90	17(5.70) 37(12.40) 7(2.30) 40(13.40) 27(9.10) 39(13.10) 14(4.70) 115(38.60) 3.40	1.21 ^*d*^ 2.70 ^*d*^ 11.35*** ^*d*^ 5.31* ^*d*^ 11.91*** ^*d*^ 0.25 ^*d*^ 2.43 ^*d*^ 0.007 ^*d*^	- - 0.15 0.11 0.16 - - -
**Telecommuting Off-site** ^a^				1.12 ^*d*^	-
Yes No Missing (%)	221(43.30) 213(41.80) 14.90	78(36.80) 65(30.70) 32.50	143(48.00) 148(49.70) 2.30		
**Average Hours of Scheduled Work per Week** ^a^				4.92 ^*d*^	-
0 to less than 30 h 30 h to less than 40 h 40 h or more Missing (%)	71(13.90) 163(32.00) 160(31.40) 22.70	25(11.80) 66(31.10) 46(21.70) 35.40	46(15.40) 97(32.60) 114(38.30) 13.80		
**Socially Distancing**				9.22** ^*d*^	0.14
Yes No Missing (%)	422(82.70) 77(15.10) 2.20	182(85.80) 19(9.00) 5.20	240(80.50) 58(19.50) 0.00		

*Note*. ^a^ Frequencies were calculated using only the subsample of participants who reported being employed at the time of the survey. ^b^ Participants were able to select more than one option. ^*c*^ *t*-test statistic was conducted. ^*d*^ Chi-square statistic was conducted. ^*e*^ Cohen’s *d* is reported for *t*-tests; *w* is reported for chi-square tests. *** *p* ≤ 0.001 ** *p* ≤ 0.01 * *p* ≤ 0.05

### Measures

The Time 2 survey (30–45 min) contained several single- and multi-item measures. We selected the most relevant (see Table 2 for complete descriptions of selected measures and internal consistency) measures for the current study to explore the different well-being profiles experienced by Canadian workers during COVID-19 and what constructs predicted profile membership. Multi-item measures capturing distress, stress, flourishing, meaning in life, impaired productivity, troublesome symptoms at work, and thriving at work were used to explore well-being profiles. It is important to note that the measure of impaired productivity is subjective, in which participants reflected on how issues (e.g., lower work quality or quantity) related to their productivity have been bothering them. Aside from demographic-related variables, several single- (i.e., financial strain, social distancing) and multi-item (i.e., family functioning, perceived vulnerability to COVID-19, sense of job security, trait resilience) measures were used to determine profile membership. The reliabilities of the multi-item measures were found to be good to excellent in this study.

**Table 2:** Description of main measures used in the current study

Construct	Scale	Citation	(Example) Item	# of Items	Scale Range	α
**Profile Indicators**						
Distress	Patient Health Questionnaire-4	Löwe et al., 2010	“Feeling nervous, anxious or on edge”	4	Not at all (1); Nearly every day (4)	0.92
Flourishing	The Psychological Well-being Scale	Diener et al., 2009	“I actively contribute to the happiness and well-being of others”	8	Strongly disagree (1); Strongly agree (7)	0.93
Impaired productivity	The Employment Absence and Productivity Scale	Lam et al., 2009	“Getting less work done”	3	None of the time (0%) (1); All the time (100%) (5)	0.85
Sense of meaning in life	Meaning in life Questionnaire	Steger et al., 2006	“Like my life is meaningful”	4	Strongly disagree (1); Strongly agree (7)	0.96
Stress	Perceived Stress Scale-4	Cohen et al., 1994	“How often have you felt that things were going your way?”	4	Never (1); Very often (5)	0.82
Thriving at work	Index of Psychological Well-Being at Work	Dagenais-Desmarais & Savoie, 2012	“I find my job exciting”	5	Disagree (0); Completely agree (5)	0.95
Troublesome symptoms at work	The Employment Absence and Productivity Scale	Lam et al., 2009	“Low energy or motivation”	4	None of the time (0%) (1); All the time (100%) (5)	0.88
**Potential Predictors of Profile Membership**						
Family functioning	Family APGAR Scale	Smilkstein et al., 1982	“I am satisfied with the way my family and I share time together”	5	Hardly ever (1); Almost always (3)	0.92
Financial strain	-	Huntley et al., 1993; Okechukwu et al., 2012; Szanton et al., 2008	“How would you describe the money situation in your household right now?”	1	Comfortable with extra (1); Cannot make ends meet (4)	-
Perceived vulnerability to COVID-19	Perceived Susceptibility Scale	Yoo et al., 2016	“Coronavirus (COVID-19) infection could happen to me”	3	Strongly disagree (1); Strongly agree (5)	0.96
Socially distancing	-	-	“Have you isolated yourself from others (i.e., social distancing) to prevent contaminating others or being contaminated with the coronavirus (COVID-19)?”	1	No (1); Yes (2)	-
Sense of job security	Crisis and/or Disaster Preparedness Scale	Fowler et al., 2007	“My job is not a secure one”	7	Strongly disagree (1); Strongly agree (7)	0.88
Trait resilience	Brief Resilience Scale	Smith et al., 2008	“I tend to bounce back quickly after hard times”	3	Strongly disagree (1); Strongly agree (5)	0.87

*Note.* α = Cronbach’s alpha

### Procedure

Wilfrid Laurier University’s Research Ethics Board approved the larger longitudinal research project this study is nested in (REB #6497). Participants were recruited in two ways: 1) (un)paid social media advertisements or b) a panel of workers managed by Qualtrics. Unpaid social media advertisements were posted on the researchers’ Facebook newsfeeds and different Facebook community groups dedicated to residents (or workers) within Canada’s provinces and territories. The panel workers were invited via a hyperlink on Qualtrics. Interested workers first completed a consent form and measures to ensure eligibility. Those eligible then completed the survey, which contained measures regarding their experiences during COVID-19. Social media participants were offered to enter a raffle for a $50 (CAD) gift card; the panel of workers received compensation set by Qualtrics.

### Data Analysis

Data preparation and descriptive statistics were conducted using SPSS (IBM Corp., 2021, V. 28.0). The study’s primary objectives were completed in MPlus (Muthén & Muthén, 1998–2017, V. 8.4).

LPA was conducted using the Robust Maximum Likelihood (MLR) estimator. The MLR estimator was optimal given our profile indicators’ continuous nature and the non-normal distribution of the impaired productivity well-being indicator (He & Fan, 2019). Random starts were used throughout the model selection process to avoid local solutions (Spurk et al., 2020). We used 5,000 sets of random starts and 500 iterations, and at least 100 of the best sets of values at the start were retained for final optimization, which surpasses recommendations (Morin et al., 2016). As recommended by Morin et al. (2016), the output was investigated to make sure that the modelling replicated the best log-likelihood at least five times. We used the following four-step procedure for model selection, which was slightly adapted from four suggestions put forward by Ram and Grimm (2009). First, the models’ relative indices of fit (AIC, BIC, SSA-BIC, CAIC) were compared. Lower values across the indices indicated a better fit. Second, we compared the likelihood ratio tests (VLMR, ALMR, BLRT). Significant ratio tests indicated that the model with *k* profiles was significantly better than the one with one less profile. Third, the models’ entropy was evaluated. A higher entropy indicated more confidence that workers were classified in one profile over the others (Weller et al., 2020). The entropy was considered while taking into account the relative indices of fit and the likelihood ratio tests. Lastly, reflecting an ongoing process during model selection, the models’ outputs were inspected for errors, out-of-bound parameters, and theoretical plausibility. A recommendation by Hamza and Willoughby (2013) was also adopted in that models with profiles containing less than 5% of the sample should be excluded. Following this recommendation, model selection halted after a model contained a profile with less than 5% of the sample.

Variables that predicted membership to the well-being profiles were explored using the auxiliary function in MPlus. The BCH function was applied for continuous variables (Bakk & Vermunt, 2016); the DCAT function was applied for categorical variables (Asparouhov & Muthén, 2021). As well-being profiles were estimated before profile membership was tested, using the BCH and DCAT auxiliary functions were optimal as they do not change the latent profiles (Asparouhov & Muthén, 2021). Although we test several potential predictors, some of the categories within these variables contained a small proportion (< 5%) of the sample. Categories found within variables containing less than 5% of the sample were either removed from these analyses or merged. Groups were only merged when it made conceptual sense (e.g., separated, divorced).

## Results

### Descriptive Statistics and Preliminary Analyses

Table 3 depicts the descriptive statistics and bivariate correlations for the well-being indicators. Few participants had missing data on the well-being indicators (0-2.86%). Most well-being indicators were fairly normally distributed, but impaired productivity was not. This was concluded based on skewness and kurtosis coefficients being greater than an absolute value of 1 (see Ramos et al., 2018) and an inspection of the measure’s Q-Q plot.

**Table 3:** Descriptive statistics and bivariate correlations for well-being profile indicators

Well-being Profile Indicators	*N*	*M*	*SD*	Skewness	Kurtosis	1.	2.	3.	4.	5.	6.	7.
1. Distress	507	1.93	0.89	0.79	-0.39	-						
2. Stress	510	2.60	0.87	0.18	-0.30	0.76	-					
3. Flourishing	506	5.20	1.13	-0.70	0.14	− 0.62	− 0.65	-				
4. Meaning in life	504	4.88	1.53	-0.75	-0.18	− 0.61	− 0.65	0.85	-			
5. Impaired productivity ^a^	407	1.68	0.80	1.54	2.43	0.62	0.53	− 0.45	− 0.37	-		
6. Troublesome symptoms at work ^a^	407	2.02	0.89	0.90	0.33	0.77	0.65	− 0.58	− 0.53	0.78	-	
7. Thriving at work ^a^	409	4.22	1.37	-0.72	-0.20	− 0.43	− 0.42	0.51	0.52	− 0.29	− 0.45	-

*Note.*^a^ Construct only measured among those employed full- and/or part-time at the time of the survey (*n* = 419). All correlations were significant at *p* < 0.001

Independent samples *t*-tests (Mann-Whitney U for impaired productivity due to its non-normal distribution) (Table 4) were conducted to explore whether social media or Qualtrics participants differed on the well-being indicators. Results showed a significant difference between the sampling sources on all well-being measures, with Qualtrics (vs. social media) participants consistently having better well-being. Whereas sampling source had a small effect on thriving at work, sampling source had a medium (distress, flourishing, impaired productivity, sense of meaning in life, stress) or large (troublesome symptoms at work) effect on other well-being indicators. As such, LPA was first conducted for each sampling source to determine whether a unique number of profiles would best represent both sources. A model with four profiles was independently found to be the best-fitting model for both subsamples. After graphing the profiles’ standardized means for each indicator, the models appeared similar across the subsamples. Due to this, we conducted LPA with the whole sample and opted to report these whole-sample findings herein.

**Table 4:** Differences between participants from social media and qualtrics on well-being profile indicators

Well-being Profile Indicator	Social Media		Qualtrics		*df*	*t or Mann-Whitney U Statistic*	*Cohen’s d*
	*M*	*SD*	*M*	*SD*			
Distress ^*a †*^	2.30	0.91	1.67	0.79	406.25	8.19	0.76
Flourishing ^*a †*^	4.82	1.17	5.47	1.02	407.23	-6.48	-0.60
Impaired productivity ^*b*^	1.95	0.86	1.54	0.73	-	12,246.00	0.53
Sense of meaning in life ^*a †*^	4.37	1.62	5.23	1.36	388.85	-6.27	-0.59
Stress ^*a*^	2.92	0.84	2.37	0.82	508	7.45	0.67
Thriving at work ^*a †*^	3.81	1.49	4.43	1.25	245.13	-4.24	-0.47
Troublesome symptoms at work ^*a*^	2.49	0.89	1.77	0.79	405	8.27	0.86

*Note*. ^*a*^ A *t*-test was conducted. ^*b*^ A Mann-Whitney U test was conducted due to a non-normal distribution. ^*†*^ Satterthwaite approximation for *t*-test results is reported due to significant Levene’s Test. M = Mean; SD = Standard deviation; *df* = Degrees of freedom. All statistics were significant at *p* < 0.001

### Source-Combined Model Selection

**Fig. 1:**
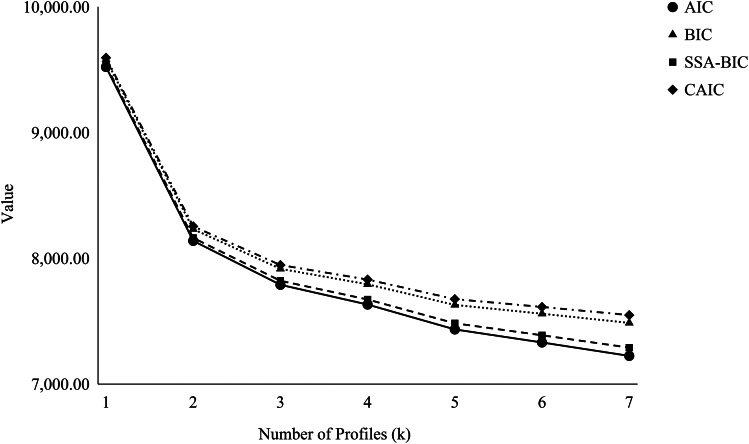
Graph of indices of fit with an increasing number of profiles (*N* = 510)

As seen in Table 5, LPA was conducted seven times using the entire dataset. As the seventh model contained a profile with less than 5% of the sample, we stopped conducting additional models. Following the first step of our adapted protocol from Ram and Grimm’s (2009) suggestions for model selection, the values for each fit indicator decreased with each model (Fig. 1), indicating a better fit (Ferguson et al., 2020; Masyn, 2013). Whereas a steep decrease was present in the two-profile model, another substantial decrease was not until the five-profile model. The five-profile model had a slight, but relatively steeper, decrease in the fit indices compared to models 3, 4, 6, and 7. This supported the retention of the five-profile model. Second, the VLMR and ALMR were not significant for most models. However, the model with five profiles had a significant VLMR and ALMR. This was in addition to the BLRT, which was significant for every model. This step supported the retention of the five-profile model. Third, and although the five-profile model had an entropy (0.85) lower than the models with fewer profiles (0.89–0.91), it exceeded the acceptable 0.80 cut-off needed to be confident that workers were well classified in one well-being profile over the others (Weller et al., 2020). As seen in Table 6, workers were highly likely to be members of their respective profiles (0.89–0.94) and not a member of the other profiles (0.00-0.10). Also considered in this step were the lower relative fit information criteria and likelihood ratio tests associated with the five-profile model. For these reasons, the five-profile model was considered the most optimal. No errors, out-of-bounds parameters, or theoretical implausibilities were found during the model-selection process. With these considerations, we retained and interpreted the five-profile model.

**Table 5:** Indices of fit for models with an increasing number of well-being profiles (*N* = 510)

Number of Profiles (*k*)	LL	FP	AIC	BIC	SSA-BIC	CAIC	VLMR	ALMR	BLRT	Entropy	Class with less than 5%
1	-4,747.81	14	9,523.61	9,582.89	9,538.45	9,596.89	-	-	-	-	No
2	-4,048.31	22	8,140.61	8,233.77	8,163.94	8,255.77	< 0.001	< 0.001	< 0.001	0.91	No
3	-3,865.55	30	7,791.09	7,918.13	7,822.90	7,948.13	0.10	0.10	< 0.001	0.86	No
4	-3,778.94	38	7,633.87	7,794.78	7,674.16	7,832.78	0.70	0.70	< 0.001	0.89	No
**5**	**-3,672.07**	**46**	**7,436.14**	**7,630.92**	**7,484.91**	**7,676.92**	**0.004**	**0.005**	**< 0.001**	**0.85**	**No**
6	-3,612.10	54	7,332.20	7,560.85	7,389.45	7,614.85	0.57	0.58	< 0.001	0.85	No
7	-3,550.74	62	7,225.47	7,488.00	7,291.21	7,550.00	0.14	0.15	< 0.001	0.85	Yes

*Note*. The model with the bolded values was the final model interpreted in the article. LL = loglikelihood; FP = the number of free parameters; AIC = Akaike Information Criteria; BIC = Bayesian Information Criteria; SSA-BIC = Sample-Size Adjusted BIC; CAIC = Consistent AIC; VLMR = Vuong-Lo-Mendell-Rubin Likelihood Ratio Test for *k*-1 profiles (H_0_) versus *k* profiles; ALMR = Lo-Mendell-Rubin Adjusted Likelihood Ratio Test for *k*-1 profiles (H_0_) versus *k* profiles; BLRT = Parametric Bootstrapped Likelihood Ratio Test for *k*-1 profiles (H_0_) versus *k* profiles

**Table 6:** Classification probabilities for the most likely latent class membership (column) by latent class (row)

	Moderately prospering profile	Prospering profile	Moderately suffering profile	Suffering Profile	Mixed profile
Moderately prospering profile	0.89	0.08	0.02	0.00	0.02
Prospering profile	0.10	0.90	0.00	0.00	0.00
Moderately suffering profile	0.04	0.00	0.91	0.01	0.04
Suffering profile	0.00	0.00	0.02	0.94	0.05
Mixed profile	0.05	0.00	0.05	0.01	0.90

### Interpretation of Well-Being Profiles

**Fig. 2:**
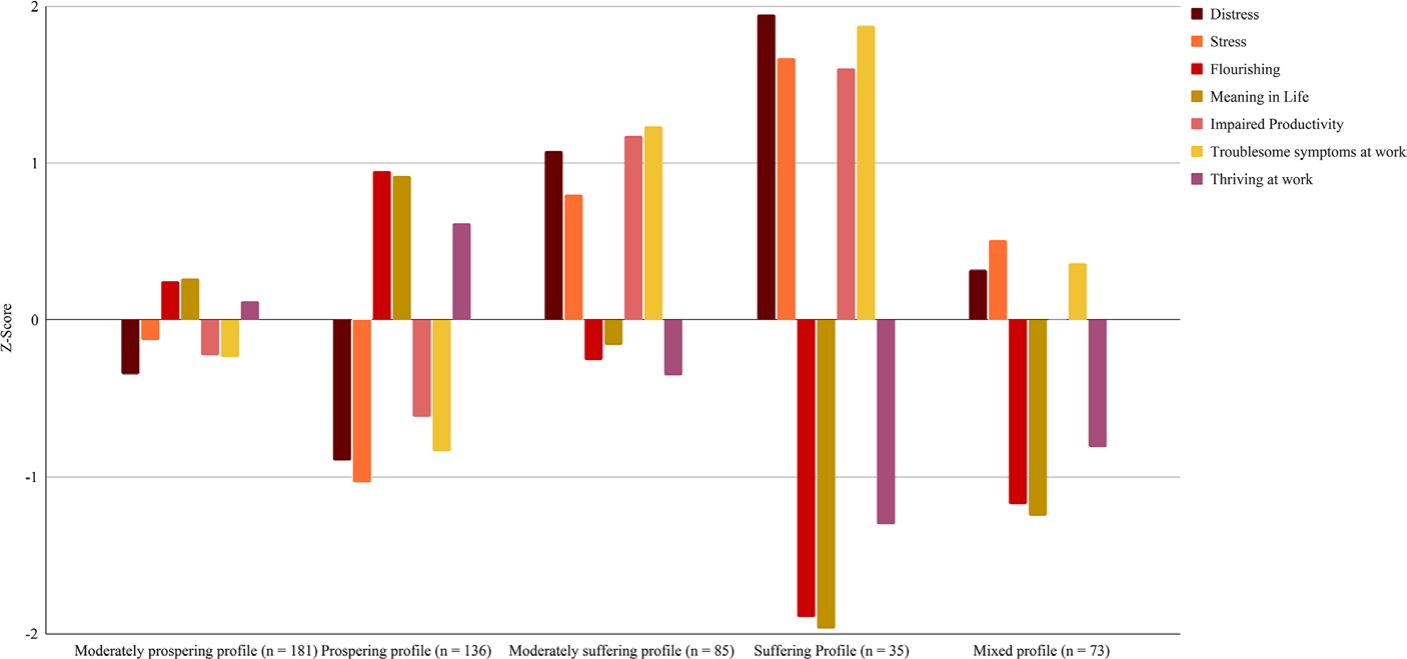
Graph of standardized means of the profiles on well-being indicators (*N* = 510) compared to the overall sample mean *Note:* Each indicator differed significantly (*p* ≤ 0.05) across profiles, except for three comparisons. For impaired productivity, mean scores were not different between the moderately prospering and mixed profiles, as well as between the moderately suffering and suffering profiles. For thriving at work, mean scores were not different between the suffering and mixed profiles

The standardized means of the five profiles on each well-being indicator are in Fig. 2. The Y-axis in this figure should be interpreted as the relative score of that profile compared to the overall sample’s score on each indicator (i.e., 0 on the center of the Y-axis). A negative z-score indicates a lower average score than the whole sample; a positive score indicates a higher average score than the whole sample. Latent profile analysis, by definition, explores multiple profiles that differ from one another. Nevertheless, we also conducted analyses using the auxiliary BCH function in MPlus to explore whether indicators differed from one another across the well-being profiles. All the indicators significantly differed from one another across the well-being profiles, except for three comparisons. For impaired productivity, two comparisons were not significant: moderately prospering and mixed profiles and the moderately suffering and suffering profiles. For thriving at work, the suffering and mixed profiles did not differ. We use terms such as “lower,” “lowest,” “higher,” and “highest” to represent best subgroups’ well-being in comparison to that of the other profiles.

The *moderately prospering* profile contained the most workers (35.49%). Workers in this profile had significantly lower scores on the indicators of negative well-being in their general lives (distress, stress) and at work (impaired productivity, troublesome symptoms at work) than the (moderately) suffering and mixed profiles. This is except for impaired productivity, however, as the lower mean score was not significantly lower than the mixed profile’s mean score. Workers in the moderately prospering profile also had significantly higher scores on indicators of positive well-being in their general lives (flourishing, meaning in life) and at work (thriving at work) than the (moderately) suffering and mixed profiles. Although having better trends across all the well-being indicators in comparison to the (moderately) suffering and mixed profiles, the mean scores in this profile were significantly lower than in the prospering profile.

The *prospering* profile contained 26.67% of the sample. The workers in the prospering profile had a similar, although even more favourable, pattern of scores on the well-being indicators as those in the moderately prospering profile. Across the profiles, the prospering profile had significantly better scores on all the well-being indicators. Thus, the prospering profile was the best well-being reality a worker could experience.

The *moderately suffering* profile contained 16.67% of the workers. Workers in this profile had significantly higher scores on the indicators of negative well-being in their general lives (distress, stress) and at work (impaired productivity, troublesome symptoms at work) than the (moderately) prospering and mixed profiles. Although having significantly worse trends across the negative well-being indicators in comparison to the (moderately) prospering and mixed profiles, these mean scores were significantly better than those in the suffering profile. This was except for impaired productivity, which was not significantly different between the two profiles. Workers in the moderately suffering profile also had significantly lower scores on indicators of positive well-being in their general lives (flourishing, meaning in life) and at work (thriving at work) than the (moderately) prospering profiles. These scores, however, were significantly higher (i.e., better) than those in the suffering and mixed profiles.

The *suffering* profile contained the least number of workers (6.86%). The workers in the suffering profile had the same pattern of results as those in the moderately suffering profile, except these workers differentiated (although not always significantly) on the well-being indicators to the greatest extent. Across the profiles, and except the non-significant difference in thriving at work between the suffering and mixed profiles, the suffering profile had significantly worse scores on all the well-being indicators. Thus, the suffering profile was the worst well-being reality a worker could experience.

Lastly, the *mixed* profile contained 14.31% of the sample. Workers in this profile had significantly higher scores on the indicators of negative well-being in their general lives (distress, stress) and at work (impaired productivity, troublesome symptoms at work) than the prospering profile. Except for impaired productivity, workers in the mixed profile had significantly worse scores on the negative well-being indicators than the moderately prospering profile. The mean scores on the negative well-being indicators were significantly lower (i.e., better) in the mixed profile than the (moderately) suffering profiles. Whereas the mean scores on the positive well-being indicators were significantly lower (i.e., worse) in the mixed profile than the moderately suffering profile, the mean scores were significantly higher (i.e., better) than the mean scores in the suffering profile. This was except for thriving at work, which did not significantly differ between the mixed and suffering profiles.

### Profile Membership

Next, we explored how factors at different ecological levels were related to belonging to the well-being profiles. A list of the tested constructs is in Table 7. The profiles with the highest proportion of workers or mean scores were reported.

**Table 7:** Association of risk and resilience factors found at different ecological levels with well-being profiles

Construct	Moderately prospering profile (1) Probability ^*a*^ *or M (S.E.)*^*b*^	Prospering profile (2) Probability ^*a*^ *or M (S.E.)* ^*b*^	Moderately suffering profile (3) Probability ^*a*^*or M (S.E.)*^*b*^	Suffering Profile (4) Probability ^*a*^ *or M (S.E.)* ^*b*^	Mixed profile (5) Probability ^*a*^ *or M (S.E.)* ^*b*^	χ^2^
Sampling Source ^a^	[2,4]	[1,3,4,5]	[2,4]	[1,2,3,5]	[2,4]	120.16***
Social media	0.46	0.12	0.54	0.81	0.55	
Qualtrics	0.55	0.88	0.46	0.19	0.45	
**Self-related Factors**						
Gender ^a^	[2,4]	[1,3,4,5]	[2,4]	[1,2,3,5]	[2,4]	89.22***
Men	0.35	0.57	0.34	0.03	0.30	
Women	0.65	0.43	0.66	0.97	0.70	
Age ^b^	[2,4] 41.23(1.12)	[1,3,4,5] 48.70(1.11)	[2,5] 38.46(1.47)	[1,2,5] 34.65(2.14)	[2,3,4] 42.97(1.59)	52.42***
Racial minority ^a^	0.23	0.12	0.08	0.09	0.09	7.33
Immigrant ^a^	0.23	0.20	0.24	0.09	0.10	8.66
Has one or more disabilities ^a^	0.13	[3,4] 0.05	[2] 0.18	[2] 0.28	0.13	12.41*
Trait resilience ^b^	[2,3,4,5] 3.35(0.07)	[1,3,4,5] 4.23(0.06)	[1,2,4] 2.81(0.09)	[1,2,3,5] 2.34(0.17)	[1,2,4] 3.05(0.10)	282.57***
**Social-related factors**						
Marital status ^a^	[4]	[4,5]	[4]	[1,2,3,5]	[2,4]	66.47***
Single	0.31	0.21	0.31	0.74	0.38	
Common law	0.15	0.12	0.13	0.11	0.19	
Married	0.46	0.56	0.50	0.09	0.33	
Separated/Divorced	0.08	0.11	0.06	0.06	0.11	
Family functioning ^b^	[2,4,5] 2.41(0.04)	[1,3,4,5] 2.85(0.04)	[2,4,5] 2.45(0.06)	[1,2,3,5] 1.80(0.10)	[1,2,3,4] 2.11(0.08)	162.30***
Children at home ^a^	[4] 0.40	0.29	0.31	[1] 0.15	0.28	10.72*
**Workplace-related factors**						
Employment status ^a^	[2]	[1,3,4,5]	[2]	[2]	[2]	52.09***
Employed full-time Employed part-time Laid off due to COVID-19 Other unemployed (e.g., retired)	0.73 0.10 0.14 0.04	0.91 0.02 0.07 0.00	0.62 0.17 0.18 0.03	0.57 0.06 0.31 0.06	0.69 0.04 0.23 0.05	
Work industry ^a^						
Education	0.12	0.17	0.19	0.08	0.18	2.99
Healthcare	0.19	0.14	0.16	0.20	0.19	1.30
Manufacturing	0.04	0.08	0.09	0.09	0.05	2.71
Services	[2,5] 0.18	[1,3] 0.09	[2,5] 0.21	[5] 0.21	[1,3,4] 0.03	15.86**
Other (e.g., automotive)	0.49	0.54	0.48	0.39	0.51	2.38
Number of scheduled hours ^a^						14.76
Less than 30 30 to less than 40 40 or more	0.15 0.46 0.38	0.16 0.33 0.52	0.33 0.38 0.29	0.12 0.40 0.48	0.13 0.51 0.35	
Telecommuting ^a^	0.48	0.55	0.52	0.42	0.52	1.68
Financial strain ^b^	[2,4] 1.91(0.06)	[1,3,4,5] 1.51(0.07)	[2,4] 2.09(0.10)	[1,2,3,5] 2.52(0.17)	[2,4] 2.09(0.12)	51.52***
Job security ^b^	[2,4] 4.26(0.12)	[1,3,4,5] 5.29(0.11)	[2,4] 4.24(0.16)	[1,2,3] 3.42(0.23)	[2] 3.88(0.22)	86.32***
**Pandemic-related factors**						
Perceived vulnerability to COVID-19 ^b^	[4] 4.10(0.07)	[4] 4.07(0.08)	[4] 4.22(0.09)	[1,2,3] 4.51(0.11)	4.23(0.10)	13.34*
Socially distancing ^a^	[3,4] 0.79	0.84	[1] 0.91	[1] 0.94	0.87	11.19*

*Note.*^a^ Profile membership estimated using the DCAT function in MPlus. ^b^ Profile membership estimated using the BCH function in MPlus. The numbers in square brackets (“[ ]”) indicates which profile(s), if applicable, the focal profile is significantly different from. χ^2^ = Chi-squared test

*** *p* ≤ 0.001, ** *p* ≤ 0.01, * *p* ≤ 0.05

The *moderately prospering* profile had a larger proportion of workers with one or more children at home. The *prospering* profile included a larger proportion of workers sampled from Qualtrics compared to other profiles. This profile had the largest proportion of workers who were: men, not disabled, married or separated/divorced. Separated and divorced workers, however, were equally as likely to be members of the mixed profile. The prospering profile included a larger proportion of workers employed full-time. Workers in the prospering profile had the highest mean age, trait resilience, positive family functioning, and sense of job security. Most of the demographic predictors of the prospering profile mirrored the sociodemographic breakdown between our sampling sources. Belonging to the *moderately suffering* profile was associated with part-time employment and working in the services industry. The *suffering* profile included a larger proportion of workers sampled from social media compared to other profiles. The suffering profile had the most workers who: identified as a woman, had one or more disability, were single. The suffering profile included a larger proportion of workers with no children at home, as well as workers who had been laid off due to COVID-19 or were experiencing other types of unemployment (e.g., retirement, on disability support). Workers in this profile had the highest mean on the measures for financial strain and perceived vulnerability to COVID-19. Most of the demographic predictors of the suffering profile mirrored the sociodemographic breakdown between our sampling sources. Interestingly, workers who were social distancing were found in a larger proportion in this profile. The *mixed* profile had the most workers in common law relationships.

## Discussion

LPA showed that the sampled Canadian workers experienced five realities: moderately prospering, prospering, moderately suffering, suffering, and mixed. Most workers experienced a (moderately) prospering well-being reality. Many constructs at the self-, social-, workplace-, and pandemic-related ecological levels were found to be predictive of workers’ well-being experience.

### Well-Being During COVID-19

In our study, we presented multiple well-being realities a diverse sample of Canadian workers were experiencing at the beginning of COVID-19. Most workers prospered to some degree (moderately prospered: 35.49%, prospered: 26.67%), indicating that most of the workers positively adapted to the pandemic. This is reminiscent of Harju et al.’s (2021) study in which most workers in the UK and France experienced a moderately positive (67%) or flourishing (8%) well-being profile during the first COVID-19 lockdown. In our study, the moderately suffering, suffering, and mixed profiles, although to different extents, were all characterized by maladaptive scores on some or all the well-being indicators. This finding (although representing one sample) suggests greater variability in the severity of suffering a worker can experience compared to the prospering they can experience. Whereas indicators of the moderately suffering or suffering profiles indicated the presence of suffering across both general and work lives among workers, in the mixed profile, workers may suffer in terms of their general well-being and some work-related well-being, but do not experience adverse nor overly favourable feelings regarding their productivity. One question stands: why were suffering workers still reporting that they were productive? One explanation is that workers may need their jobs to afford necessities (e.g., shelter, food), especially during COVID-19. The risk of losing one’s job due to low performance could lead to unattainable basic needs. Another explanation is that maintained productivity was expected by employers irrespective of the changing pandemic conditions. For example, working mothers in the US described a workload intensification following pandemic-related disruptions (Zanhour & Sumpter, 2022). Without adequate mental health resources, it seems likely that while workers’ self-reported productivity was not impacted negatively, their well-being at work and general lives could have been.

The findings provide a unique insight into the dynamic process of resilience. Aside from the mixed profile, workers prospered (or suffered) similarly across the explored well-being domains. This is seen as workers’ well-being was affected similarly in their work lives as in their general life. These results may inform well-being-related policies as stakeholders (e.g., policymakers, mental health organizations) can develop interventions that simultaneously address general and work-related well-being. Although the suffering profile is the smallest well-being profile of the five, it provides rich information regarding the proportion of workers not adapting well to COVID-19. Stakeholders can use this information to prioritize person-centered resources for workers suffering the most.

### Risk and Resilience Factors During the Pandemic

**Fig. 3:**
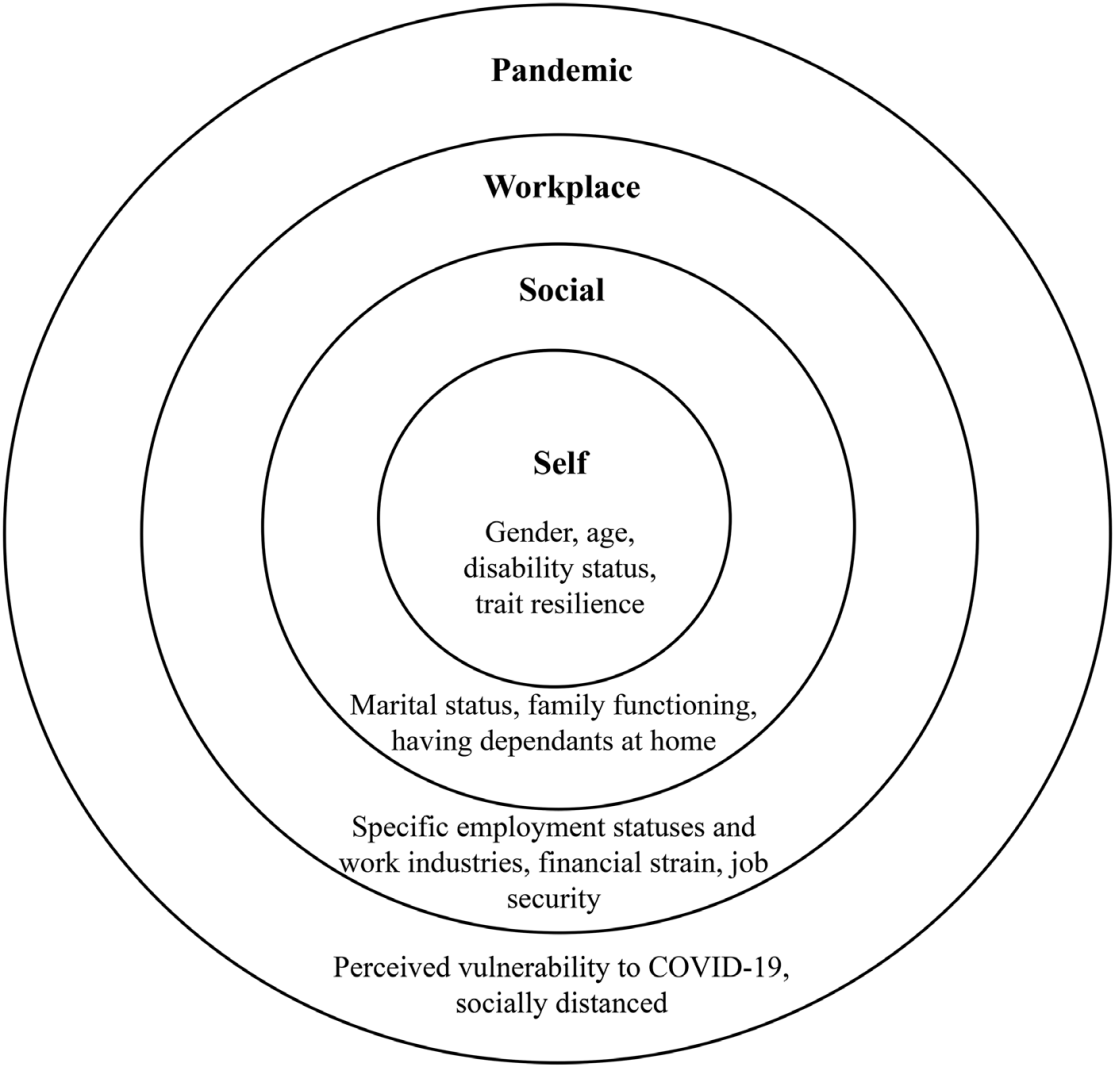
The ecological nesting of the significant predictors of well-being profile membership

Many factors at various ecological levels (Fig. 3) significantly predicted membership to the well-being profiles. Reminiscent of articles contextualized before the COVID-19 pandemic or after its beginning in various samples, and reflecting self-related factors, we found that identifying as a woman (e.g., Hansen & Blekesaune, 2022), being younger (e.g., Carney et al., 2021), having one or more disabilities (e.g., Turner et al., 2006), and lower trait resilience (e.g., Hu et al., 2015) were associated with less odds of being in a prospering profile. These findings highlight which workers public (e.g., policymakers, mental health organizations) and workplace (e.g., employers, lower/middle management) stakeholders can focus their attention. Extending from research on the benefits of peer support groups (e.g., Strand et al., 2020; Walker & Bryant, 2013), mental health organizations, for example, can develop support groups for these workers.

Across the social-related factors, our findings indicate that the presence of (positive) familial or romantic connections is related to prospering, whereas no (or negative) familial or romantic connections is related to suffering. One exception to this trend is marital status, in which contrasting relationship statuses were associated with the same well-being profile. These findings are thought to reflect the complexity of social relationships. With the exception that divorced and separated workers were equally likely to be in the prospering and mixed profiles, consider how separated, divorced, and married workers were as likely to experience the prospering profile compared to other profiles. Thomas et al. (2017) posit that the relationship between marital links and well-being depends on the relationship’s quality. Marriage quality may act as a mechanism, thus explaining why those with(out) a romantic partner in the present study were prospering. However, research is needed to disentangle if this effect is due to potential confounding variables. Whereas our findings regarding family functioning are reminiscent of literature conducted on diverse samples during public health crises (e.g., Brooks et al., 2018; Song et al., 2022), our findings regarding parenthood are nuanced. Nelson et al. (2014) describe that the mixed relationship between parenthood and well-being could be explainable by confounding variables. Here, we show that working parents were most represented in the moderately prospering profile, whereas the suffering profile included more workers with no children. This reflects Nelson et al.’s (2014) proposition that parents may be unhappy to the extent they experience greater negative emotions related to parenthood, whereas they may be happy when experiencing more positive emotions and meaning in life related to parenthood.

Regarding workplace-related factors, workers (moderately) prospering were employed full-time or had the highest sense of job security. Workers (moderately) suffering were those employed part-time, working in the services industry, laid off due to COVID-19, experiencing other types of unemployment (e.g., retirement, on disability support), or with the highest financial strain. A common trend exists across these factors in that two dimensions of precarious employment, employment insecurity and income inadequacy (Kreshpaj et al., 2020), were linked with more suffering. Suffering workers had the lowest average job security score (Table 7). These findings are aligned with The Law Commission of Ontario (n.d.), who reported that retail and cashier workers (operationalized as “services” workers here) were the second-highest precarious occupational group. In our study, workers in the services industries were found to be (moderately) suffering. Our findings, like other articles (e.g., Allan et al., 2021), suggest that working in precarious employment sectors can impact workers’ well-being. In line with previous research showing how best to implement initiatives addressing precarious employment (Gunn et al., 2022), governments should: (1) provide general support, (2) federally regulate and enforce core labour standards, and (3) collaborate with other stakeholders (e.g., employers, non-governmental organizations, unions) (Gunn et al., 2022).

Lastly, perceived vulnerability to COVID-19 and social distancing – as pandemic-related factors - significantly predicted profile membership. The suffering profile included workers with the highest perceived vulnerability to COVID-19. This finding is reminiscent of previous literature showing that higher risk or exposure to a pathogen is related to poorer well-being (e.g., Shaukat et al., 2020). Falco et al. (2021) found that safety systems (e.g., quality and effectiveness of organizations’ policies, procedures, or interventions in improving COVID-19-related safety outcomes), communication, and (participating in) decision-making buffered the relationship between Italian workers’ perceived risk of work-related infection and emotional exhaustion. The implementation of such workplace factors may be a method employers can use to mitigate workers’ suffering amid pandemics.

Although associated with benefits, the implementation of telecommuting needs to be done carefully. Telecommuting can lead to more isolation and less communication among individuals in an organization (Rogers, 2022). As discussed below, social distancing may have negative psychological effects, thus warranting the implementation of additional interventions. Here, workers who were socially distancing were more represented in the suffering profile. Although beneficial for safeguarding physical health, social distancing may bring negative psychological impacts. Hwang et al. (2020) share a large cost associated with essential quarantine and social distancing interventions: loneliness. Experienced loneliness was related to several negative mental health outcomes before COVID-19. For example, Lee et al. (2019) reported that high loneliness in a sample of adults in California was related to greater cognitive complaints (e.g., forgetfulness, distractibility), depression, anxiety, and perceived stress, as well as poorer resilience, optimism, mental well-being, and wisdom. Thus, interventions are needed to mitigate social distancing policies’ adverse psychological effects. Policymakers, for example, could subsidize mental health services (e.g., therapy).

### Positive Psychology and the Utilization of Person-Centered Analyses

In this study, we show the utility of applying third-wave positive psychology when marrying theories regarding social determinants of mental health (Alegría et al., 2018), resiliency (Pan & Chan, 2007), complete well-being (Keyes, 2005; Westerhof & Keyes, 2010), the ecological systems that workers are nested in (Bronfenbrenner, 1986; Jason et al., 2016), and precarious work (Kreshpaj et al., 2020). Drawing on these diverse theories provided an unparalleled aid as each theory provided a unique but synergetic perspective that helped explain the complex effects of the pandemic. It helped us understand the well-being realities Canadian workers were experiencing, as well as who was prospering and suffering.

The use of person-centered approaches is still in its infancy in positive psychology. We show the utility of LPA in identifying patterns that are unobtainable by variable-centered approaches: profiles describing diverse, multiple well-being realities. Not only did we find the most common well-being realities workers were experiencing at the beginning of the pandemic, but the smaller, negative, realities that need more intervention. The ability to detect such realities are not only important to positive psychologists, but also to stakeholders aiming to create interventions that aid those who are, or are at risk of, suffering.

### Limitations

First, the findings are not generalizable given that some workers (e.g., in some industries and provinces/territories) are underrepresented and could not be included in the analyses exploring profile membership. In a similar vein, these findings were determined using data from Canadian workers, leaving unexplored the well-being realities that exist for workers in other countries. Second, prospering workers may be more likely to participate in such empirical studies, potentially explaining why the (moderately) prospering profiles contained the most workers. Third, well-being measures were selected as profile indicators that were psychometrically sound, but decisions were made as to the measures (and how many) were used to determine latent profiles. These decisions may have influenced the number of profiles and their shapes. Fourth, LPA provides a snapshot of workers’ well-being at a single time. What is left unanswered is the trajectories characteristic of workers’ well-being before and after that moment. Further, the data was collected at the beginning of the pandemic, so the findings represent workers’ well-being at a single moment during a turbulent period during COVID-19. Exploring well-being realities early in the pandemic is important because it may set the tone for workers’ overall adaptation later in the pandemic. However, the findings are not characteristic of workers’ well-being during the full length of the pandemic.

## Conclusion

Given the diverse well-being realities different workers experienced during pandemics, it is not advised to assume that all workers experience a singular reality. Although some were found to be suffering, these findings suggest that many workers were adapting well at the beginning of COVID-19. A better understanding of the well-being realities and the factors associated with prospering and suffering can contribute to interventions that improve the well-being of workers suffering while continuing to support those prospering.

## Data Availability

As per our consent form, for access to the data and the syntax, please e-mail the corresponding author.
